# Combined Deficiency of the Melanocortin 5 Receptor and Adenosine 2A Receptor Unexpectedly Provides Resistance to Autoimmune Disease in a CD8^+^ T Cell-Dependent Manner

**DOI:** 10.3389/fimmu.2021.742154

**Published:** 2021-11-16

**Authors:** Trisha McDonald, Fauziyya Muhammad, Kayleigh Peters, Darren J. Lee

**Affiliations:** ^1^ Dean McGee Eye Institute, Department of Ophthalmology, University of Oklahoma Health Sciences Center, Oklahoma City, OK, United States; ^2^ Department of Microbiology and Immunology, University of Oklahoma Health Sciences Center, Oklahoma City, OK, United States

**Keywords:** autoimmune uveitis, A2a adenosine receptor (A2Ar), melanocortin 5 receptor, Treg - regulatory T cell, CD8 T cell

## Abstract

Regulatory immunity that provides resistance to relapse emerges during resolution of experimental autoimmune uveitis (EAU). This post-EAU regulatory immunity requires a melanocortin 5 receptor (MC5r)-dependent suppressor antigen presenting cell (APC), as shown using a MC5r single knock-out mouse. The MC5r-dependent APC activates an adenosine 2A receptor (A2Ar)-dependent regulatory Treg cell, as shown using an A2Ar single knock-out mouse. Unexpectedly, when MC5r^-/-^ post-EAU APC were used to activate A2Ar^-/-^ post-EAU T cells the combination of cells significantly suppressed EAU, when transferred to EAU mice. In contrast, transfer of the reciprocal activation scheme did not suppress EAU. In order to explain this finding, MC5r^-/-^A2Ar^-/-^ double knock-out (DKO) mice were bred. Naïve DKO mice had no differences in the APC populations, or inflammatory T cell subsets, but did have significantly more Treg cells. When we examined the number of CD4 and CD8 T cell subsets, we found significantly fewer CD8 T cells in the DKO mice compared to WT and both single knock-out mice. DKO mice also had significantly reduced EAU severity and accelerated resolution. In order to determine if the CD8 T cell deficiency contributed to the resistance to EAU in the DKO mice, we transferred naïve CD8 T cells from WT mice, that were immunized for EAU. Susceptibility to EAU was restored in DKO mice that received a CD8 T cell transfer. While the mechanism that contributed to the CD8 T cell deficiency in the DKO mice remains to be determined, these observations indicate an importance of CD8 T cells in the initiation of EAU. The involvement of CD4 and CD8 T cells suggests that both class I and class II antigen presentation can trigger an autoimmune response, suggesting a much wider range of antigens may trigger autoimmune disease.

## Introduction

Ocular inflammation, uveitis, can cause permanent damage to the light-gathering structures of the eye, resulting in permanent blindness. Uveitis is the third leading cause of blindness in countries with access to cataract surgery, with an incidence between 25.6 – 122 cases per 100,000 a year, and a prevalence of 69 - 623 cases per 100,000 (1–3). Active uveitis patients experience transient or permanent vision loss occurs with an estimated 12.5% going on to develop glaucoma (4). Autoimmune uveitis patients experience relapsing and remitting inflammation, with 33% of anterior uveitis cases becoming chronic (5). A better understanding of the immunobiology that contributes to relapsing and remitting intraocular inflammation has the potential to develop novel more effective treatments for autoimmune uveitis.

Experimental autoimmune uveitis (EAU) is the most widely used mouse model of human autoimmune uveitis (6). In C57BL/6J mice, resolution of EAU occurs at 75-90 days following immunization for EAU without further relapse (7–9), and at this point (post-EAU) regulatory immunity is found in the spleen (10). This post-EAU regulatory immunity provides resistance to relapse and suppresses EAU when transferred to recipient EAU mice (10–13). A critical component of post-EAU regulatory immunity is expression of the adenosine 2A receptor (A2Ar) for activation and differentiation of Treg cells, and expression of the melanocortin 5 receptor (MC5r) on the post-EAU suppressor antigen presenting cell (APC) (11, 12). While A2Ar-expressing post-EAU Tregs and MC5r-expressing APC are not required for resolution of EAU (11, 14), it has been shown that depletion of all Foxp3^+^ Treg cells before resolution of EAU prevents resolution (15). Therefore, the resolution of EAU and induction of regulatory immunity that provides resistance to relapse occur independently of one another. As such, a better understanding of the melanocortin-adenosinergic pathway is needed.

It is well documented that CD8^+^ T cells have a role in uveitis through observations in rodent models of EAU (16–19), and analysis of aqueous humor from uveitis patients (20, 21). MC5r transcript expression has been reported in CD8^+^ T cells from human PBMCs (22) but protein expression was not confirmed and the role of MC5r in this cell subset has not been further examined. A2Ar has been extensively studied in the field of cancer biology, and has a suppressive role on CD8^+^ T cells, as tumor evasion by the immune system is observed with blockade of A2Ar on CD8^+^ T cells (23, 24). However, the role of MC5r and A2Ar have not been examined on CD8^+^ T cells in the context of autoimmune uveitis.

In this report, we demonstrate that the melanocortin-adenosinergic pathway is more nuanced than was previously understood and involves more cell types than previously reported. In addition to the involvement in the induction and activation of post-EAU regulatory immunity, the melanocortin-adenosinergic pathway has an additional role in the disease phase of EAU that requires CD8^+^ T cells for the induction of EAU.

## Methods

### Mice

All mouse procedures described in this study were approved by the University of Oklahoma Health Sciences Center Institutional Animal Care and Use Committee (OUHSC IACUC) and all mouse study methods were carried out in accordance with the relevant guidelines approved by the OUHSC IACUC. C57BL/6J mice and adenosine 2A receptor knock-out (A2Ar^-/-^) mice were purchased from Jackson Laboratories. Melanocortin 5 receptor knockout mice (MC5r^-/-^) mice on a C57BL/6J background were a generous gift from Roger D. Cone (Oregon Health Sciences, Portland, Oregon). A2Ar^-/-^ MC5r^-/-^ double knock-out (DKO) mice were bred in the Dean McGee Eye Institute vivarium, and the genotype was confirmed by PCR in the DMEI Genotyping Core.

### Experimental Autoimmune Uveoretinitis

EAU was induced in mice according to the previously described immunization protocol (13). An emulsion of complete Freund’s adjuvant (CFA) with 5 mg/mL desiccated *M. tuberculosis* (Difco Laboratories, Detroit, MI) and 2 mg/ml interphotoreceptor retinoid binding protein (peptides 1-20) (IRBP) (Genscript, Piscataway, NJ) was used to immunize mice for EAU. A volume of 100 µl was injected subcutaneously at two separate sites in the lower back along with an intraperitoneal injection of 0.3 µg pertussis toxin. Fundus examinations using a slit lamp microscope occurred every 3-4 days to monitor the severity of retinal inflammation over the course of EAU. To examine the retina, the iris was dilated using 1% tropicamide, the cornea was numbed with 0.5% proparacaine, and the cornea was flattened with a glass coverslip in order to examine the retina. The severity of EAU was scored on a 5-point scale, as previously described (25), using the clinical signs of observable infiltration and vasculitis in the retina. Both eyes were scored and the higher score was taken to represent that mouse for that day, and the average score for the group was calculated.

### *In Vitro* Stimulation

Spleens were collected into 5% FBS in RPMI supplemented with 10 μg/ml Gentamycin (Sigma), 10 mM HEPES, 1 mM Sodium Pyruvate (BioWhittaker), Nonessential Amino Acids 0.2% (BioWhittaker) and made into a single cell suspension that was depleted of red blood cells using RBC lysis buffer (Sigma, St Louis, MO). The spleen cells were resuspended in serum free media (SFM) and IRBP was added at 50 μg/mL for 48 hours at 37°C and 5% CO_2_ to reactivate antigen specific T cells. SFM consisted of RPMI-1640 with 1% ITS+1 solution (Sigma) and 0.1% BSA (Sigma). Following the reactivation, supernatants were collected and analyzed and/or cells were collected for adoptive transfer into recipient mice.

In some experiments antigen presenting cells (APC) and T cells were cultured from different strains. APC were collected by incubating splenocytes in SFM at 37°C and 5% CO_2_ for 90 minutes in tissue culture plates, washed twice, and adherent cells were scraped off of the plastic in ice cold SFM, and plated at 4 x 10^5^ cells per well. CD3 enriched T cells were obtained from post-EAU spleens using a CD3 enrichment column (R&D Systems), added to the sorted APC at 8 x 10^5^ cells per well with 50 μg IRBP peptide, and cultured at 37°C 5% CO_2_ for 48 hours. After 48 hours, T cells and APC were collected, and washed in PBS. Following the reactivation, cells were collected for adoptive transfer into recipient mice.

### Adoptive Transfer

Cultured cells described above were collected under sterile conditions washed with sterile PBS, and resuspended in sterile PBS. Mice were injected with 1 x 10^6^ activated post-EAU cells in PBS into the tail vein. Following the adoptive transfer, the mice were immunized for EAU as described above.

### Cytokine Analysis

Cell culture supernatants were assayed using the mouse Th1/Th2/Th17/Th22/Treg 18-multiplex procartaplex kit, (Invitrogen, Vienna, Austria) to assay the culture supernatants. The Multiplex plate was analyzed with Bio-Rad plate reader (Bioplex system, Hercules, CA). The assay was performed according to manufacturer’s instructions.

### Flow Cytometry

Mouse spleen cells were washed with PBS with 1% BSA (staining buffer), blocked with mouse IgG in staining buffer, then stained with conjugated antibodies. Antibodies used were anti-CD11b (clone M1/70, Biolegend, San Diego, CA), anti-Ly-6C (clone HK1.4, Biolegend), anti-Ly-6G (clone 1A8, Biolegend), anti-F4/80 (clone BM8, eBiosciences, San Diego, CA), anti-MHCII (clone M5/114.15.2, Biolegend), anti-CD4 (clone RM4-5, Biolegend), anti-PD-1 (clone 29F.1A12, Biolegend), anti-Foxp3 (clone FJK-16s, eBiosciences), anti-Tbet (clone 4B10, Biolegend), and anti-RORγt (clone AFKJS-9, Biolegend). Prior to anti-Foxp3, anti-Tbet, and anti-RORγt staining, the cells were fixed and permeabilized.

Stained cells were analyzed in the Oklahoma Medical Research Facility (OMRF) Flow Cytometry Core Facility on a BD LSRII (BD Biosciences) or the DMEI Ocular Immunobiology Core on a 4-laser Aurora (Cytek Biosciences, Fremont, CA). When the Aurora was used, unmixing was done using SpectroFlo Software (Cytek) and data was analyzed using FlowJo Software (Tree Star, Inc., Ashland, OR).

### Statistics

Statistical significance between maximum EAU scores was determined using nonparametric Mann-Whitney U test between groups of mice. Two-way ANOVA was also used to assess significant changes in the tempo of disease between the groups of treated EAU mice with post-test Bonferroni comparison analysis. Statistical significance was determined when P ≤ 0.05 (two-sided). All statistical tests were conducted in R v3.5.1 and statistical analysis for mouse experiments were analyzed with Graphpad Prism software.

## Results

### The Melanocortin-Adenosinergic Pathway Is More Complicated Than Previously Observed

**Figure 1:**
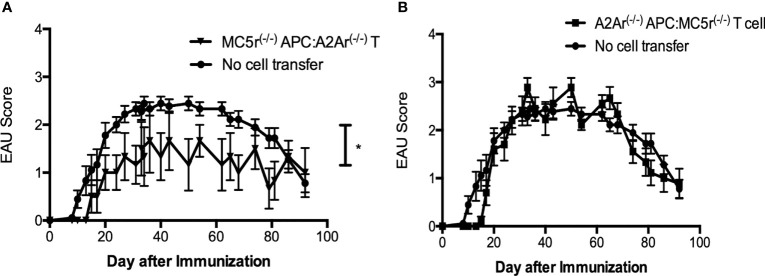
Activation requirements of post-EAU regulatory macrophages and T cells in MC5r^-/-^ and A2Ar^-/-^ mice. APC and T cells from post-EAU MC5r^-/-^ and A2Ar^-/-^ mice were collected. APC and T cells were cultured *in vitro* with IRBP and adoptively transferred to recipient mice immunized for EAU. The solid line with closed circles are EAU control mice that did not receive an adoptive transfer of spleen cells (no cell transfer, n = 18). Post-EAU MC5r^-/-^ APC were used to activate post-EAU A2Ar^-/-^ T cells and transferred to a recipient mouse and immunized for EAU (**A**, solid line with inverted triangles, n = 6). Post-EAU A2Ar^-/-^ APC were used to activate post-EAU MC5r^-/-^ T cells and transferred to a recipient mouse and immunized for EAU (**B**, solid line with squares, n = 10). Disease was monitored every 3-4 days from the time of immunization through resolution. Each experiment consisted of 4-5 mice, and each experiment was repeated 2-4 times. Significant suppression of disease was observed compared to EAU mice that received no cell transfer (*P< 0.05), determined by two-way ANOVA with Bonferroni post-test.

The aim of this study is to further understand the role of the melanocortin-adenosinergic pathway in the induction of autoimmune uveitis. We have previously reported that the melanocortin-adenosinergic pathway is necessary for the induction of post-EAU regulatory immunity that provides resistance to relapse (11, 14, 26, 27). Our previous work specifically showed that expression of the melanocortin 5 receptor (MC5r) on the post-EAU antigen presenting cell (APC) is required for activation of post-EAU regulatory T cells that express the adenosine 2A receptor (A2Ar). We therefore reasoned that a post-EAU MC5r^-/-^ APC would be unable to activate a post-EAU A2Ar^-/-^ T cell. However, when transferred to a recipient mouse immunized for EAU we observed a significant suppression of disease compared to EAU mice that received no cell transfer (**Figure 1A**). When the reciprocal activation scheme was used, we found that post-EAU A2Ar^-/-^ APC used to activated post-EAU MC5r^-/-^ T cells were unable to suppress disease in recipient mice (**Figure 1B**). These unexpected observations suggested the interplay between APC and T cells through the melanocortin-adenosinergic pathway is more complicated than previously understood. We next asked what the role of this pathway is during the induction of disease using a double knockout (DKO) mouse with the MC5r^-/-^ A2Ar^-/-^ genotype.

### The DKO Mice Have No Difference in Suppressor APCs, More Tregs, and Are Resistant to Disease

**Figure 2:**
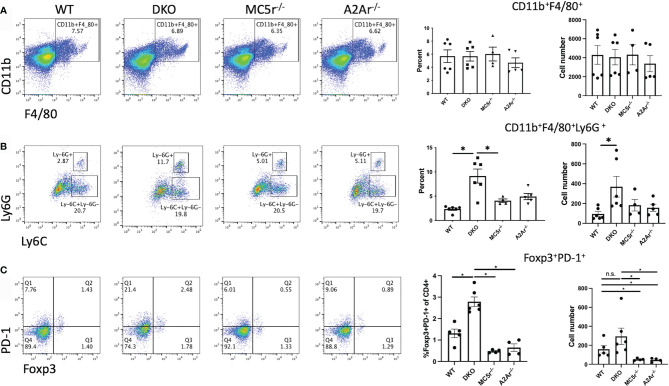
Comparison of APC and Tregs in naïve DKO with WT, MC5r^-/-^, and A2Ar^-/-^ mice. Spleens from naïve WT (N=6), DKO (N=6), MC5r-/- (N= 4), and A2Ar-/- (N = 5) were collected and stained for CD11b, F4/80, Ly6G, Ly6C, CD4, PD-1, and Foxp3. Representative pseudocolor dot plots and the mean ± SEM for each mouse are shown for CD11b and F4/80 **(A)**, Ly6G and Ly6C **(B)**, and PD-1 and Foxp3 **(C)**. The Ly-6G and Ly6C panels are gated on CD11b^+^F4/80^+^ cells, and PD-1 and Foxp3 panels are gated on CD4^+^ cells. Each experiment consisted of 1-2 mice, and each experiment was repeated 2-3 times. Significance was assessed by nonparametric Mann-Whitney U test. Statistical significance (P ≤ 0.05) is designated by *, or not significant (n.s.).

We first asked if the APC compartment is different in DKO mice compared to the single knockout (SKO) mice. Spleens from naïve WT, DKO, MC5r^-/-^, and A2Ar^-/-^ were collected and stained for CD11b, F4/80, Ly6G, and Ly6C as we have done before (14). We observed a similar number of CD11b^+^F4/80^+^ macrophages between each of the four strains of mice (**Figure 2A**). However, we did observe significantly more CD11b^+^ F4/80^+^ Ly6G^+^ Ly6C^lo^ macrophages in DKO mice compared to WT, MC5r^-/-^, and A2Ar^-/-^ mice (**Figure 2B**), but did not observe a significant difference in the abundance of CD11b^+^ F4/80^+^ Ly6G^+^ MHCII^+^ macrophages between the WT, DKO, MC5r^-/-^, and A2Ar^-/-^ mice (**Supplementary Figure 1**). Because the CD11b^+^ F4/80^+^ Ly6G^+^ Ly6C^lo^ macrophages have previously been demonstrated to be the suppressor macrophage population at the resolution of disease that activates post-EAU Tregs (11, 14), we asked if there were more PD-1^+^ Foxp3^+^ Tregs, the Tregs we previously identified as post-EAU Tregs that suppress disease (27). Comparison of the Treg compartment between WT, DKO, MC5r^-/-^, and A2Ar^-/-^ mice revealed that DKO mice had significantly more PD-1^+^Foxp3^+^ Tregs than the WT, MC5r^-/-^, and A2Ar^-/-^ mice (**Figure 2C**).

**Figure 3:**
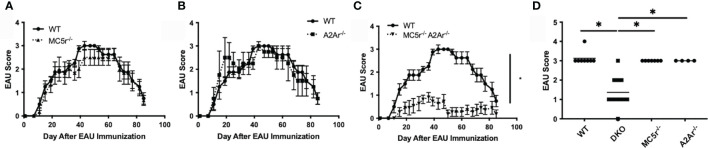
The course of EAU in WT, MC5r^-/-^, A2Ar^-/-^, and DKO mice. WT mice (n = 8), MC5r^-/-^ (n = 7), A2Ar^-/-^ (n = 4), and DKO (n = 11) mice were immunized for EAU and evaluated from the time of immunization every 3 - 4 days for clinical signs of uveitis. The course of disease MC5r^-/-^ (**A**, dashed line with triangles), A2Ar^-/-^ (**B**, dashed line with squares), and DKO (**C**, dashed line with inverted triangles) is shown with WT mice (solid line with circles). The severity of disease is indicated by the maximum score of each mouse over the entire course of disease **(D)**. Each experiment consisted of 1-4 mice, and each experiment was repeated 2-3 times. Significance was assessed by two-way ANOVA with Bonferroni post-test for EAU scores over the course of disease and nonparametric Mann-Whitney U test for maximum scores. Statistical significance (P ≤ 0.05) is designated by *.

We next asked what the effect of the MC5r and A2Ar deficiency in DKO mice has on EAU. WT, DKO, MC5r^-/-^, and A2Ar^-/-^mice were immunized for EAU and monitored through the course of disease. As we previously observed, MC5r^-/-^ and A2Ar^-/-^ mice showed no significant change in EAU tempo and severity (**Figures 3A, B, D**). However, we observed a significant reduction in the severity and course of disease in DKO mice (**Figures 3C, D**). These observations reveal an unexpected role of these receptors on the induction of disease that is in contrast with what we have observed in relation to the induction of regulatory immunity that provides resistance to disease.

### DKO Mice Have More Tregs and Th1 Cells During the Disease Phase

**Figure 4:**
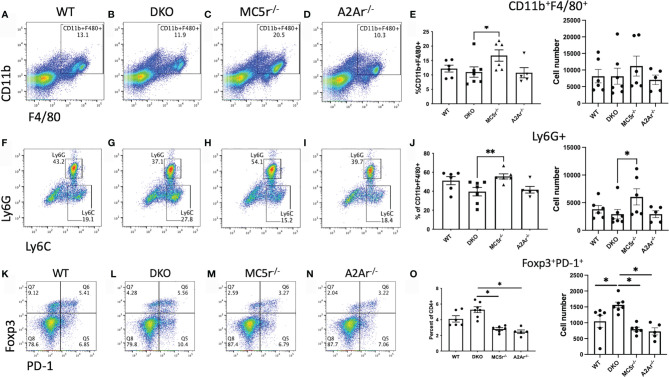
Comparison of APC and Tregs at the onset of EAU in DKO with WT, MC5r^-/-^, and A2Ar^-/-^ mice. Spleens from EAU onset (day 24) WT (n=6), DKO (n=7), MC5r^-/-^ (n= 6), and A2Ar^-/-^ (n= 5) were collected and stained for CD11b, F4/80, Ly6G, Ly6C, CD4, PD-1, and Foxp3. Representative pseudocolor dot plots and the mean ± SEM for each mouse are shown for CD11b and F4/80 **(A–E)**, Ly6G and Ly6C **(F–J)**, and PD-1 and Foxp3 **(K–O)**. The Ly-6G and Ly6C panels are gated on CD11b+F4/80+ cells, and PD-1 and Foxp3 panels are gated on CD4+ cells. Each experiment consisted of 1-2 mice, and each experiment was repeated 2-3 times. Significance was assessed by nonparametric Mann-Whitney U test. Statistical significance (*P ≤ 0.05 or **P ≤ 0.01) is designated by * and **.

We next sought to understand the mechanism providing resistance to disease in the DKO mice. We first asked if the APC compartment is different in DKO mice compared to the single knockout (SKO) mice at the onset of disease. Spleens from naïve WT, DKO, MC5r^-/-^, and A2Ar^-/-^ were collected and stained for CD11b, F4/80, Ly6G, and Ly6C as we have done before (14). We observed a significant decrease in the abundance of CD11b^+^F4/80^+^ macrophages in MC5r^-/-^ mice compared to DKO mice (**Figures 4A–E**). We also observed a significant decrease in the abundance of CD11b^+^ F4/80^+^ Ly6G^+^ Ly6C^lo^ macrophages in DKO mice compared to MC5r^-/-^ mice (**Figures 4F–J**). However, we did not observe a significant difference in the abundance of CD11b^+^ F4/80^+^ Ly6G^+^ MHCII^+^ macrophages between the WT, DKO, MC5r^-/-^, and A2Ar^-/-^ mice (**Supplementary Figure 1**).

**Figure 5:**
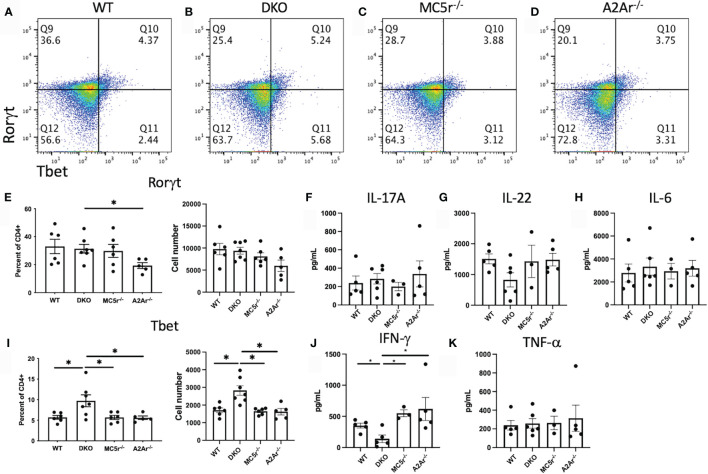
T cell response at the onset of EAU in WT, MC5r^-/-^, A2Ar^-/-^, and DKO mice. Spleens from WT (n = 4-6), DKO (n = 6-7), MC5r-/- (n = 3-6) and A2Ar^-/-^ (n = 5) were collected, restimulated with IRBP, and assayed for Treg, Th17, and Th1 profiles from at the onset (day 24) of disease. Representative pseudocolor flow cytometry dot plots are shown for Rorgt and Tbet expression from the spleen **(A–D)** with the mean ± SEM for all mice. The mean ± SEM is shown for Rorgt **(E)** IL-17A **(F)**, IL-22 **(G)**, IL-6 **(H)**, Tbet **(I)**, IFN-g **(J)**, and TNF-a **(K)**. Significance was determined by Mann-Whitney U test. Statistical significance (P ≤ 0.05) is designated by *.

We next asked what the T cell response was at the onset of disease. Spleens were collected, restimulated, and assayed for Treg, Th17, and Th1 profiles. Flow cytometry staining revealed more Foxp3^+^PD-1^+^ Tregs (**Figures 4K–O**) and Rorγt^+^ T cells (**Figures 5A–E**) in DKO mice compared to A2Ar^-/-^ mice in the CD4^+^ compartment. However, the Th17-associated cytokines, IL-17A, IL-22, and IL-6 were not significantly different between the four genotypes (**Figures 5E–H**). The transfer of post-EAU splenocytes to EAU mice resulted in a significant suppression of EAU in recipient mice (**Supplementary Figure 2**), suggesting a dominant regulatory phenotype in the DKO mice. We observed a significant increase in the number of Tbet^+^ T cells in DKO mice compared to WT, MC5r^-/-^, and A2Ar^-/-^ mice among CD4^+^ T cells (**Figure 5I**). However, DKO mice compared to WT, MC5r^-/-^, and A2Ar^-/-^ mice, produced significantly less IFN-γ and production of TNF-α was not significantly different (**Figures 5J, K**). These observations demonstrate that T cell polarization is disrupted in DKO mice, such that the characteristic Treg, Th1, and Th17 transcription factors are expressed, but the characteristic inflammatory cytokine is not significantly elevated.

### DKO Mice Have a Reduced CD8 T Cell Compartment

**Figure 6:**
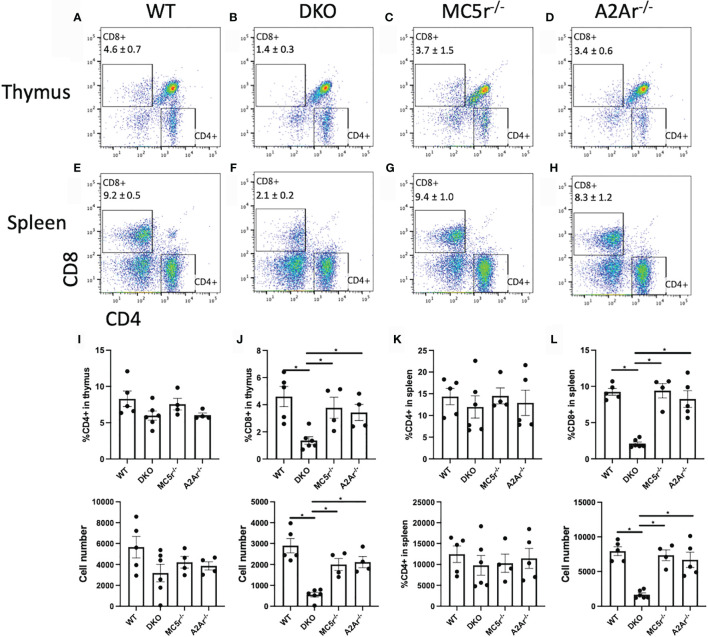
Comparison of CD4 and CD8 T cells in WT, MC5r^-/-^, A2Ar^-/-^, and DKO mice. The spleens and thymus of naïve WT (n = 4-5), DKO (n = 6), MC5r^-/-^ (n = 4) and A2Ar^-/-^ (n = 4-5) mice were harvested and stained for CD8 and CD4. Representative pseudocolor flow cytometry dot plots are shown for CD8 and CD4 expression from the thymus **(A–D)** and spleen **(E–H)** with the mean ± SEM for all mice for thymic cells **(I, J)** and spleen cells **(K, L)**. Each experiment consisted of 1-2 mice, and each experiment was repeated 2-3 times. Significance was assessed by nonparametric Mann-Whitney U test. Statistical significance (*P ≤ 0.05) is designated by *.

We next pursued an explanation for the observation that DKO had a significantly greater abundance of Tbet^+^ cells but significantly reduced IFN-γ production. Because CD8^+^ T cells can also produce IFN-γ (28, 29), we asked if there was a reduced number of CD8^+^ T cells in the DKO mice. We observed a significant reduction in CD8^+^ T cells in the spleen and thymus of DKO mice compared to WT, MC5r^-/-^, and A2Ar^-/-^ mice (**Figures 6A–H, J, L**). In contrast, the number of CD4^+^ T cells between all four genotypes was not significantly different (**Figures 6A–H, I, K**). These observations suggest that a defect in the development of CD8^+^ T cells occurs in the DKO mice and could explain why these mice are resistant to EAU.

### CD8 T Cells From WT Mice Allow for EAU Susceptibility in DKO Mice

**Figure 7:**
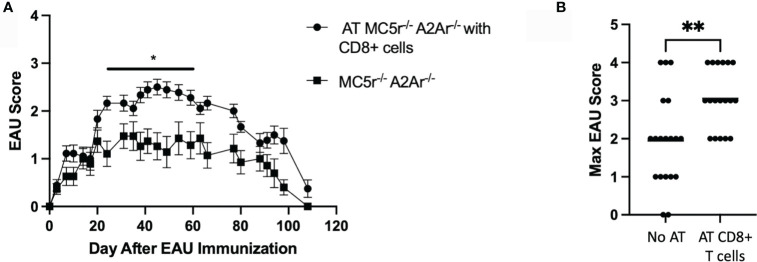
The course of EAU in DKO mice following transfer of WT CD8^+^ T cells. EAU was induced in DKO mice that received an adoptive transfer of WT CD8^+^ T cells two days before immunization (solid line with circles, n = 18) or EAU DKO mice that did not receive a cell transfer (solid line with squares, n = 19). Mice were immunized for EAU and evaluated from the time of immunization every 3 - 4 days for clinical signs of uveitis **(A)**. The severity of disease is indicated by the maximum score of each mouse over the entire course of disease **(B)**. Each experiment consisted of 4-7 mice, and each experiment was repeated 3-4 times. Significance was assessed by two-way ANOVA with Bonferroni post-test for EAU scores over the course of disease and nonparametric Mann-Whitney U test for maximum scores. Statistical significance (*P ≤ 0.05 or **P ≤ 0.01) is designated by * and **.

We next tested if restoring the CD8^+^ T cell compartment in DKO mice is sufficient to eliminate resistance to EAU. EAU scores of mice that received a transfer of WT CD8 T cells before immunization for EAU were significantly elevated compared to DKO mice immunized for EAU that did not receive a cell transfer (**Figure 7A**). Additionally, the maximum EAU scores were not significantly different between the DKO EAU mice that received a CD8 T cell transfer and DKO EAU mice that received no cell transfer (**Figure 7B**). These observations demonstrate that a WT CD8^+^ T cell compartment is necessary to overcome the resistance to EAU observed in DKO mice.

## Discussion

In this report, we sought to further define the role of MC5r and A2Ar in the induction of post-EAU regulatory immunity and determine the role of MC5r and A2Ar during the course of disease. Our observations reveal a more complicated role for MC5r expression of APC and A2Ar on Treg cells in the induction of post-EAU regulatory immunity that remains to be investigated. This observation drove us to create a DKO mouse to investigate the role of a deficiency of both MC5r and A2Ar in the same mouse. We unexpectedly found that DKO mice were resistant to disease. The DKO mice had a similar APC compartment, but elevated Tregs, and reduced CD8^+^ T cells. We found that a transfer of CD8^+^ T cells from WT mice was sufficient to overcome the disease resistance in DKO mice. These observations demonstrate the complexity of MC5r and A2Ar on the pathogenesis of EAU and in the induction of regulatory immunity, and shows the importance of CD8^+^ T cells in susceptibility to autoimmune disease. Based on this work we suggest that CD8^+^ T cells that are dependent on MC5r and A2Ar expression suppress Tregs and are necessary to induce autoimmune uveitis (**Supplementary Figure 3**).

The RORγt T cell transcription factor expression in DKO mice was elevated in the DKO compared to A2Ar^-/-^ mice. However, the observation that cytokine production was no different for IL-17A, IL-22, IL-6, or TNF-α suggests that while the transcriptional profile was altered, there was no polarization towards Th17 in the DKO mice. We did observe a significantly increased number of Tregs in the DKO compared to MC5r^-/-^ and A2Ar^-/-^ mice, which suggests the resistance to disease may be due to an increased abundance of Tregs. This is not unexpected, given previous observations that Tregs are necessary for resolution of disease (15). We further observed the Tregs in the DKO mice did suppress EAU when transferred to WT EAU mice, which showed the DKO Tregs are functionally suppressive.

The observation that the Th1 transcription factor, Tbet, was expressed in significantly more CD4^+^ T cells in DKO mice compared to WT, MC5r^-/-^, and A2Ar^-/-^ mice suggested DKO mice were skewed to a Th1 polarization. However, the significant reduction of IFN-γ production suggested the DKO mice were not skewed toward a Th1 phenotype. We further explored this possibility by quantifying the CD8^+^ T cells in the spleen and thymus and found DKO mice had significantly less CD8^+^ T cells in both tissues. In order to determine if the lack of CD8^+^ T cells in DKO mice was the cause of disease resistance, CD8^+^ T cells were transferred from WT mice, and we observed disease in mice that received the transfer. These observations demonstrate the resistance to EAU in the DKO mice is dependent on CD8^+^ T cells. However, further investigation of the role of the melanocortin-adenosinergic pathway in CD8^+^ T cells is needed to understand the melanocortin-adenosinergic role in CD8^+^ T cells during the induction of EAU. A potential mechanism that could explain the requirement for A2Ar and MC5r on CD8^+^ T cells to induce EAU could be that the limited number CD8^+^ T cells from these mice are deficient in their effector functions. Since significantly fewer CD8^+^ T cells were observed in the thymus of DKO mice, this suggests there is a defect in thymic development of the CD8^+^ cells in the DKO mice. There may be an insufficient activation signal that allows for CD8^+^ T cells to undergo positive selection, a defect with negative selection in CD8^+^ T cells, or the deficiency in A2Ar and MC5r blocks expression of the CD8 co-receptor. This possibility will be investigated in future studies. However, if this is the mechanism for the reduction of CD8^+^ T cells in DKO mice, this would be a novel mechanism that highlights a nuance between CD4 and CD8 T cell development.

Others have demonstrated the importance of minimally activated CD8^+^ T cells in uveitis in rats and mice (16–19), and CD8^+^ T cells are present in eyes of uveitis patients (20, 21). While it is clear that effector CD8^+^ T cells are involved in the inflammatory immune response, the connection with activation of these cells with the melanocortin-adenosinergic pathway is paradoxical. CD8^+^ regulatory T cells are an essential component of anterior chamber associated immune deviation (ACAID) (30, 31), and minimally activated CD8^+^ T cells in EAU are suppressive (32). These observations suggest there may be some overlap between the induction of post-EAU regulatory immunity and the ACAID response, and provides some possible insight into the paradoxical observation that a MC5r and A2Ar deficiency results in reduced susceptibility to EAU. While additional work is needed to better understand this observation, it suggests that MC5r and A2Ar have a role in the development of CD8^+^ T cells, and this population may have a role in the induction of post-EAU regulatory immunity. A future question to address is if the defect is intrinsic to bone-marrow derived cells, which could be addressed with bone marrow chimeras.

These observations also illustrate the disconnect between the induction of post-EAU regulatory immunity that provides resistance to relapse and resolution of disease with resolution of EAU. A mechanism of ocular immune privilege is the induction of systemic regulatory immunity against ocular antigen. We and others have found this regulatory immunity to emerge at resolution of uveitis (10, 26, 27, 33). However, we have found that resolution of uveitis can occur without induction of post-EAU regulatory immunity (12, 14). Therefore, this report adds to the body of evidence that resolution of uveitis does not necessarily provide the regulatory immunity that provides resistance to relapse. The unfortunate consequence of the independent induction of this regulatory immunity and resolution is that patients may experience remission, but are susceptible to relapse. It is possible that the 33% of uveitis patients that become chronic (5) do so because of the lack of regulatory immunity that provides resistance to relapse. Our previous observations that stimulation of the melanocortin-adenosinergic pathway only induces regulatory T cells in a subset of patients (14, 26, 34), supports this hypothesis based on our animal model observations.

While ocular immune privilege may have evolved to protect the delicate light-gathering tissues of the eye, it is not perfect. It is thought the selection pressure to maintain vision had a selective evolutionary advantage in the gathering of food and detection of predators (35–37). In the context of evolutionary pressure, elimination of lethal pathogens would have taken precedence over preservation of vision. Therefore, another explanation for our paradoxical results could be that a compensatory mechanism for survival exists in the DKO mice that also provides resistance to EAU. As such, in some cases the evolutionary pressure for survival comes at the cost of loss of ocular immune privilege, followed by the loss of vision.

## Data Availability Statement

The raw data supporting the conclusions of this article will be made available by the authors, without undue reservation.

## Author Contributions

All experiments, analysis, and experimental design of this work was done by DL, FM, and TM. The conceptual design of this work and the writing of this manuscript was a collaborative effort between DL and KP. All authors contributed to the article and approved the submitted version.

## Funding

This work was supported by National Institutes of Health/National Eye Institute grants EY021725 (P30), EY024951 (DL), and in part by an unrestricted Research to Prevent Blindness grant (New York, NY, USA).

## Conflict of Interest

The authors declare that the research was conducted in the absence of any commercial or financial relationships that could be construed as a potential conflict of interest.

## Publisher’s Note

All claims expressed in this article are solely those of the authors and do not necessarily represent those of their affiliated organizations, or those of the publisher, the editors and the reviewers. Any product that may be evaluated in this article, or claim that may be made by its manufacturer, is not guaranteed or endorsed by the publisher.
