# Supplementary figures and images for: Combined Deficiency of the Melanocortin 5 Receptor and Adenosine 2A Receptor Unexpectedly Provides Resistance to Autoimmune Disease in a CD8+ T Cell-Dependent Manner
Source: Front Immunol. 2021 Nov 16;12:742154. doi: 10.3389/fimmu.2021.742154 (PMC8634946; doi:10.3389/fimmu.2021.742154)

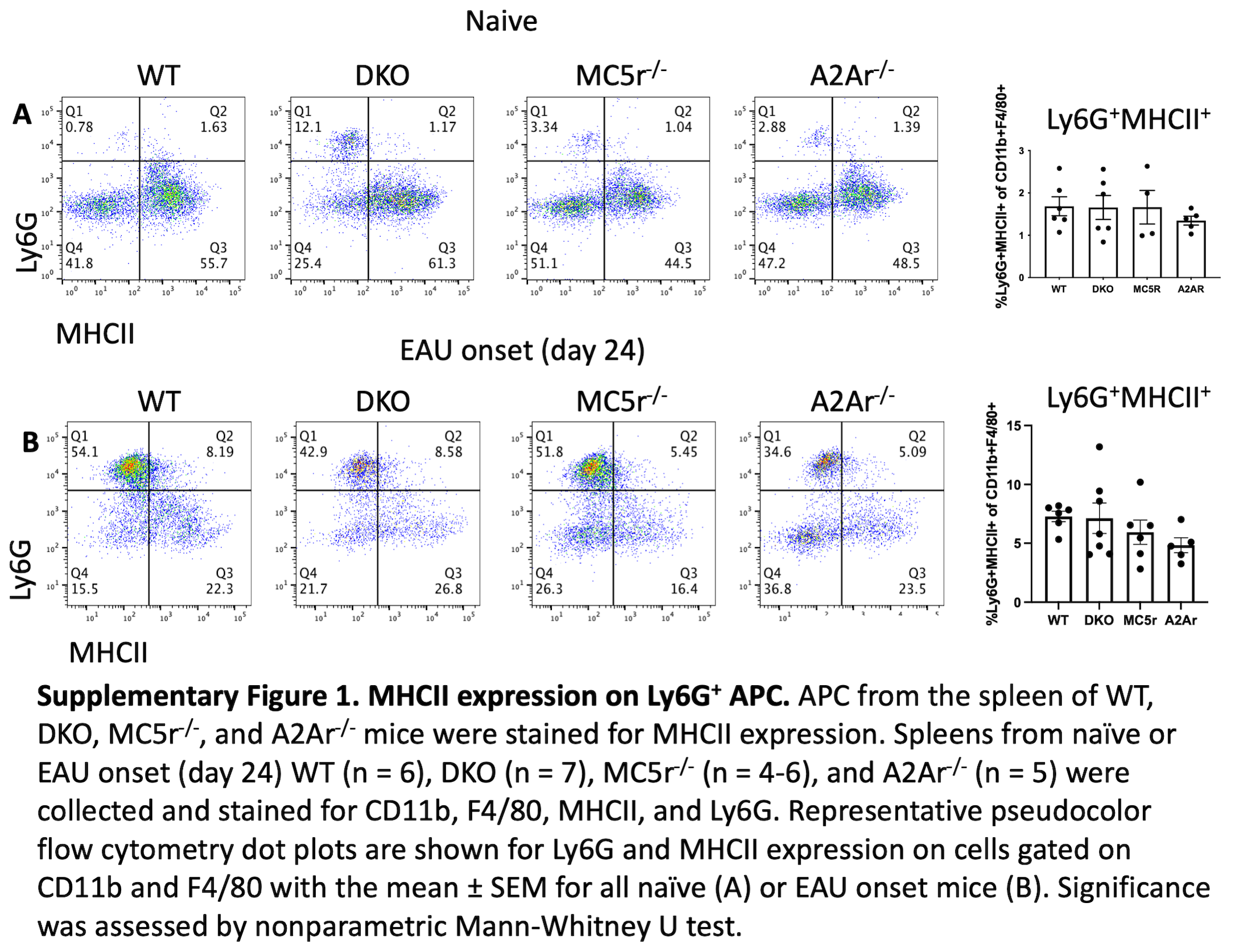

Supplement: Supplementary file 1 [file Image_1.tiff]

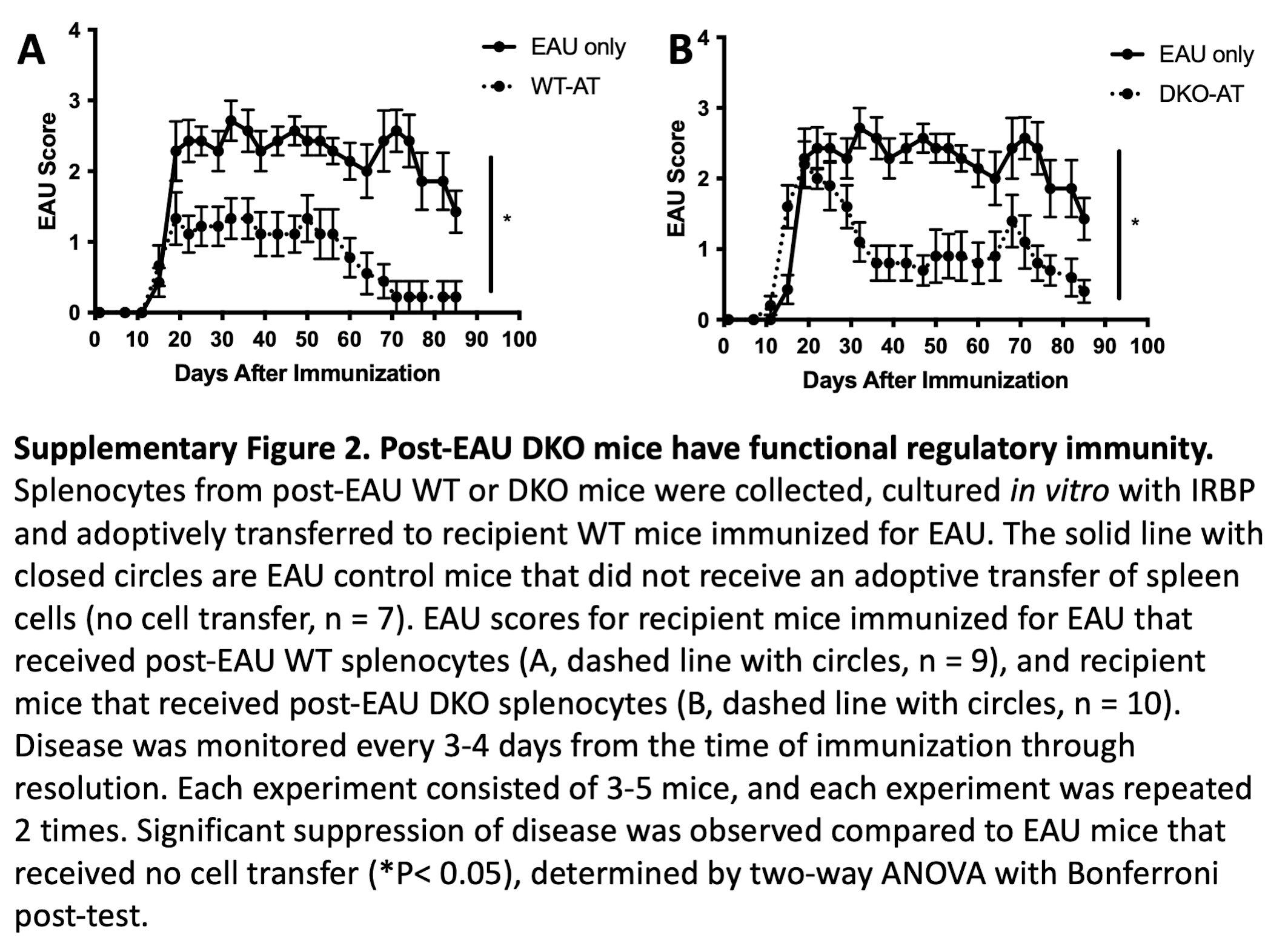

Supplement: Supplementary file 2 [file Image_2.tiff]

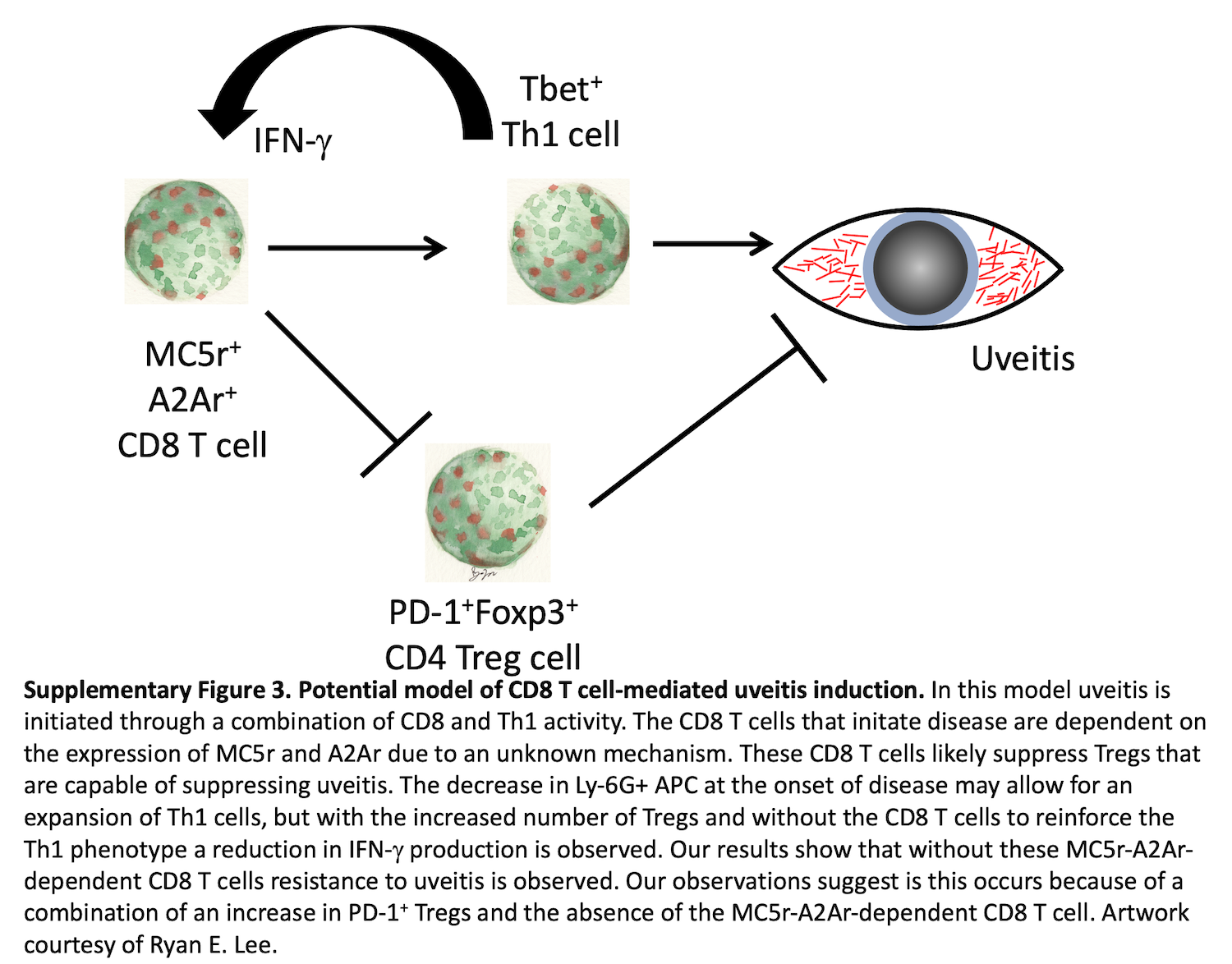

Supplement: Supplementary file 3 [file Image_3.tiff]
